# Linc-NSC affects cell differentiation, apoptosis and proliferation in mouse neural stem cells and embryonic stem cells in vitro and in vivo

**DOI:** 10.1007/s00018-024-05224-0

**Published:** 2024-04-14

**Authors:** Lili Guo, Dan Zou, Wenqiao Qiu, Fan Fei, Lihua Chen, Wenjin Chen, Huan Xiong, Xinda Li, Yangyang Wang, Mingjun Gao, Jianwei Zhu, Jin Zhang, Yunsen He, Mou Gao, Ruxiang Xu

**Affiliations:** 1 Department of Neurosurgery, Sichuan Provincial People’s Hospital, University of Electronic Science and Technology of China, Chengdu, China; 2 Chinese Academy of Sciences Sichuan Translational Medicine Research Hospital, Chengdu, 610072 China; 3 Department of Neurosurgery, Chinese PLA General Hospital, Beijing, 100853 China

**Keywords:** Stem cells, Transplantation, Genetic manipulation, Tumorigenicity

## Abstract

**Background:**

Stem cell therapy is a promising therapeutic strategy. In a previous study, we evaluated tumorigenicity by the stereotactic transplantation of neural stem cells (NSCs) and embryonic stem cells (ESCs) from experimental mice. Twenty-eight days later, there was no evidence of tumor formation or long-term engraftment in the NSCs transplantation group. In contrast, the transplantation of ESCs caused tumor formation; this was due to their high proliferative capacity. Based on transcriptome sequencing, we found that a long intergenic non-coding RNA (named linc-NSC) with unknown structure and function was expressed at 1100-fold higher levels in NSCs than in ESCs. This finding suggested that linc-NSC is negatively correlated with stem cell pluripotency and tumor development, but positively correlated with neurogenesis. In the present study, we investigated the specific role of linc-NSC in NSCs/ESCs in tumor formation and neurogenesis.

**Methods:**

Whole transcriptome profiling by RNA sequencing and bioinformatics was used to predict lncRNAs that are widely associated with enhanced tumorigenicity. The expression of linc-NSC was assessed by quantitative real-time PCR. We also performed a number of in vitro methods, including cell proliferation assays, differentiation assays, immunofluorescence assays, flow cytometry, along with in vivo survival and immunofluorescence assays to investigate the impacts of linc-NSC on tumor formation and neurogenesis in NSCs and ESCs.

**Results:**

Following the knockdown of linc-NSC in NSCs, NSCs cultured in vitro and those transplanted into the cortex of mice showed stronger survival ability (P < 0.0001), enhanced proliferation(P < 0.001), and reduced apoptosis (P < 0.05); the opposite results were observed when linc-NSC was overexpressed in ESCs. Furthermore, the overexpression of linc-NSC in ECSs induced enhanced apoptosis (P < 0.001) and differentiation (P < 0.01), inhibited tumorigenesis (P < 0.05) in vivo, and led to a reduction in tumor weight (P < 0.0001).

**Conclusions:**

Our analyses demonstrated that linc-NSC, a promising gene-edited target, may promote the differentiation of mouse NSCs and inhibit tumorigenesis in mouse ESCs. The knockdown of linc-NSC inhibited the apoptosis in NSCs both in vitro and in vivo, and prevented tumor formation, revealing a new dimension into the effect of lncRNA on low survival NSCs and providing a prospective gene manipulation target prior to transplantation. In parallel, the overexpression of linc-NSC induced apoptosis in ESCs both in vitro and in vivo and attenuated the tumorigenicity of ESCs in vivo, but did not completely prevent tumor formation.

**Supplementary Information:**

The online version contains supplementary material available at 10.1007/s00018-024-05224-0.

## Introduction

The transplantation of stem cells (SCs) presents a promising avenue for the advancement of tissue engineering and regenerative medicine applications [1, 2]. Currently, the available SCs include neural stem cells (NSCs), embryonic stem cells (ESCs), and mesenchymal stem cells (MSCs). Our previous research demonstrated that NSCs exhibit a reduced tumorigenic potential and a more limited capacity for differentiation when compared to MSCs and ESCs. However, NSCs have the ability to differentiate into all cell types required for the central nervous system (CNS). The advancement of NSC therapy is limited due to the dependence of NSC transplantation on the quantity of the delivered cells, which does not necessarily ensure optimal outcomes [3]. Challenges such as extensive grafted-cell death, reduced proliferation, increased apoptosis, and the diminished differentiation capacity of transplanted SCs in comparison to their native counterparts persist and cannot be resolved solely by augmenting cell count. In addition, the potential tumorigenicity of transplanted ESCs/MSCs at the graft site appears to impede the efficacy of ESCs/MSCs transplantation [4–6]. Therefore, we sought to investigate the key molecules that determine whether SCs are tumorigenic or not.

Gene manipulation techniques, such as the cellular reprogramming of NSCs via the regulation of c-Myc, high-mobility group box 1 (HMGB1), interleukin 6, mutated estrogen receptor transgene, or PEP-1-SOD1, have been shown to promote proliferation, differentiation into neuronal lineage, and migration [7–10]. Chen et al. conducted a study in which NSCs were cultured in an alginate scaffold; this method resulted in increased survival rates [11]. Nevertheless, the overall efficacy of these techniques remains limited, particularly following transplantation.

Long non-coding RNA (lncRNA) is a class of RNA molecules that exceed 200 nucleotides in length and do not encode proteins. However, lncRNAs are known to play a crucial role in regulating gene expression at multiple levels, including the epigenetic, transcriptional, and post-transcriptional levels [12]. Typically, lncRNAs are categorized as intergenic (lincRNA) or intronic lncRNA based on their location. Guttman et al. demonstrated that numerous lincRNAs in embryonic stem cells (ESCs) can interact with transcription factors associated with pluripotency, such as Oct4, Nanog, and Sox2 [13]. The impact of lincRNAs on the expression of pluripotency-related genes has been observed through transaction. In another study, Loewer et al. revealed a significant increase in the expression levels of certain lincRNAs in iPSCs when compared to ESCs [14]. The activation of these lincRNAs has been shown to enhance the reprogramming efficiency of iPSCs, and they have been identified as direct targets of pluripotency-related transcription factors (Oct4, Nanog, and Sox2), thus indicating a close association between specific lincRNAs and the pluripotency of iPSCs. The identification of an increasing number of lincRNAs, such as lincRNA-p21, has clearly demonstrated their regulatory role in angiogenesis [15]. The inhibitory effect of human lincRNA-RoR on p53-mediated cell cycle arrest and apoptosis has been established [14], while LINC01225 has been shown to promote the proliferation, invasion, and migration of gastric cancer through the Wnt/β‐catenin signaling pathway [16]. Despite the vast number of lincRNAs identified, their specific functions have been defined in less than 1% of cases [17].

In a previous study, we demonstrated that the transplantation of NSCs or induced neural stem cells (iNSCs) is less tumorigenic than that of ESCs or MSCs, even when the number of cells is insufficient for survival [18]. To further investigate this phenomenon, we utilized mRNA microarray analysis to generate differential expression profiles between ESCs/MSCs and NSCs/iNSCs. These data indicated that the expression of lincRNA (chr16: 4047, Supplementary File 1) located on chromosome 16 in NSCs/iNSCs is 1100 times greater than that of ESCs/iPSCs. Because of the striking evolutionary conservation of lincRNA sequence and CNS-specific expression pattern, we named this linc-NSC. Gene Ontology analysis and Kyoto Encyclopedia of Genes and Genomes pathway analysis suggests that linc-NSC may be negatively correlated with stem cell pluripotency and a positive correlation with neurogenesis. Thus, in this study, we sought to investigate the role of linc-NSC in tumorigenicity, pluripotency, and neurogenesis by manipulating its expression in NSCs and ESCs. These investigations are particularly relevant given the pressing need to improve transplant survival rates.

## Materials and methods

### Animals

C57BL/6 mice (male, 6 weeks old, 25–30 g) were purchased from the Biotechnology Corporation of Dashuo (Chengdu, China) and housed in pathogen-free facilities. All the animal raising and handling protocols were approved by the Animal Care and Use Committee of the Hospital of the University of Electronic Science and Technology and Sichuan Provincial People’s Hospital.

### Isolation and culture of mouse NSCs

NSCs were harvested from the cerebral cortices of 14-day-old C57BL/6 mouse embryos. Fetal brain tissue was acquired by laparotomy under aseptic conditions. Brains were harvested under a stereomicroscope, meninges and blood vessels were carefully stripped, and the cerebral hemisphere was separated. Then, we used microsurgical forceps to cut the cerebral hemisphere tissue and digest it with 0.175% trypsin (DNase 50:1) for 10 min. An equal volume of culture medium containing 10% (v/v) fetal bovine serum (FBS; Gibco) was added to terminate the digestion. Cells were dispersed by pipetting repeatedly to create a single-cell suspension with culture medium and filtered through a 40 µm sieve. Fresh medium was supplied every 2–3 days and cells were passaged every 5–7 days.

### Mouse embryonic fibroblasts (MEFs) isolation and ESCs cultures

MEFs were harvested from the cerebral cortices of 14-day-old C57BL/6 mouse embryos. The head and internal organs were removed, and the torso was minced and dispersed in 0.25% trypsin–EDTA (DNase 50:1) for digestion for 15 min. After repeated gentle blowing and beating the tissue, the cell suspension was neutralized by an equal volume of MEF complete medium (DMEM/F12,10% FBS,1% penicillin–streptomycin). Finally, cells were filtered with a 30 μm cell strainer. The cell suspension was centrifuged (1000 rpm for 5 min) and resuspended with complete culture medium, and then inoculated in a 100 mm culture dish coated with 0.1% gelatin, recorded as P0. All cells were cultured in a 37 °C, 5% CO_2 _incubator. Mouse ESCs were purchased from Oricell (Cyagen Biosciences, Guangzhou, China, MUBES-01001) and cultured in mouse ESC growth medium (Oricell, MUBES-90011) on plates precoated with gelatin solution and MEFs as feeder cells.

### Microarray analysis

Total RNA was isolated from the cells of each group using TRIzol reagent (Invitrogen). RNA expression profiling was then performed using the Agilent mouse lncRNA + mRNA microarray V2.0 platform. The arrays were scanned by the Agilent G2565CA Microarray Scanner. Agilent Feature Extraction software (version 11.0.1.1) was used to analyze acquired array images. Quantile normalization and subsequent data processing were performed using the GeneSpring GX v11.5.1 software package (Agilent Technologies). Differentially expressed genes were identified by fold change filtering.

### Cell transfection and the generation of stable cell lines

NSCs were cultured in complete media. Stable linc-NSC knockdown cells were generated by infection with lentivirus-based shRNA. A linc-NSC knockdown lentivirus as well as a control lentivirus were purchased from Hanheng Biotechnology (Shanghai, China). The lentiviral-based vector pHBLV-U6-Scramble-ZsGreen-Puro was used to express shRNA (shRNA construct: GATCCGAGCTGACTTCTTAGGGCGATGGTATTCAAGAGATACCATCGCCCTAAGAAGTCAGCTCTTTTTTG) while a lentivirus-expressing non-target shRNA control (5´-GATCCGTTCTCCGAACGTGTCACGTAATTCAAGAGATTACGTGACACGTTCGGAGAATTTTTTC) was used as a control. NSCs (P2) were transfected according to the supplier’s instructions. Viral stocks were added at 10 multiplicity of infection (MOI), and cell-virus suspensions were centrifuged in ultra-low-adhesion round bottom 24-well plates at 37 °C for 24 h before they were resuspended in 2 mL of medium and transferred to a 6-well dish. After 5 days, puromycin was added at 0.1 µg/mL to select transfected cells.

Mouse ESCs were cultured in mouse ESC growth medium on plates that were precoated with gelatin solution and MEFs as feeder cells. Stable ESCs were transduced with the linc-NSC overexpression lentivirus and the linc-NSC overexpression control lentivirus and then cultured. The linc-NSC overexpression lentivirus, as well as the control lentivirus, were purchased from Hanheng Biotechnology (Shanghai, China). The overexpression lentivirus vectors were used by VP001-CMV-MCS-EF1-ZsGreen-T2A-Puro and ESCs were transfected according to the supplier’s instructions (Hanheng Biotechnology, Shanghai, China). Virus stocks were added at 400 MOI; 48 h after viral infection, puromycin (2 µg/mL) was added for screening. During the screening process, if there were too many dead cells, the puromycin-containing culture medium was replaced, and the colonies were grown and passaged (1:5). After three days of screening, we replaced the culture medium containing puromycin (1 µg/mL), and continued to culture for two generations for subsequent experiments.

Transfection efficiency was determined by calculating the proportion of GFP-positive cells via fluorescent microscopy (Zeiss AX10). Cells were used for experiments within five passages post- transfection. For all the following experiments, the cells in each group had a good growth state.

### Quantitative real-time PCR

The quantification of mRNA levels of pluripotency and neurogenesis genes were analyzed by qPCR and normalized to β-actin (Actb). Total RNA was extracted from the cultured cells (R701-01, Vazyme). Reverse transcription was performed using HiScript III All-in-one RT SuperMix (R333-01, Vazyme) according to the manufacturer’s protocol. The qPCR was used to determine linc-NSC knockdown in the shRNA group, the overexpression group, and the control group and was performed with Taq pro Universal SYBR qPCR Master Mix (Ref Q712-02, Vazyme). The qPCR reactions were run on a Bio‐Rad real‐time qPCR system (Bio‐Rad). Quantitative results for each sample were determined by the 2−∆∆Cq method by PCR amplification. Primers are listed in Supplementary File 2.

### Proliferation assay

Cell viability was measured by the CCK-8 assay (A311, Vazyme). In brief, cells were seeded in 96-well plates at a density of 10,000 cells/well in 100 µL of culture medium and fed in the incubator overnight. CCK-8 solution (10 µL per 100 µL medium) was then added to each well. Subsequently, we incubated the plates for 2 h in a 5% CO_2_ atmosphere, and the absorbance at 450 nm was measured using a microplate reader. Cell viability was represented as a proportion (%) of the control.

### Flow cytometry

Cells were detached by Accutase (Sigma-Aldrich) for 5–10 min, fixed with 4% paraformaldehyde at room temperature for 30 min, and then permeabilized with 70% ethanol for at least 2 h at 4 °C using a Cell Cycle and Apoptosis Analysis Kit (Beyotime) and Apoptosis Detection Kit (BD Biosciences). The quadrants of these plots indicated live cells (AnV−/PI−;), early-stage apoptosis (AnV + /PI−;), or late-stage apoptosis (AnV + /PI +). Each sample was analyzed by flow cytometry using a Guava easyCyte (Millipore) equipped with a 488-nm blue laser. Dead cells and debris were excluded by gating on forward scatter and pulse-width profiles.

### Stereotaxic injections

C57BL/6 mice (male, 6–8 weeks old) were allocated randomly to experimental groups: a shRNA group, a shRNA-control group, an overexpression group, and an overexpression control group. Animals were anesthetized with 1% pentobarbital sodium and maintained under deep anesthesia using 2% isofluorane. Mice were fixed in a brain stereotaxic apparatus (RWD Life Science) and depilated. An iodophor was used for disinfection and a paved sterile sheet was used. The site of injection was located 1 mm lateral to the medial sagittal suture, 5.5 mm rostral to the lambda, and 2.0 mm in depth under the dura mater. Injections were performed with a 10 μL microinjector at a speed of 0.5 μL/minute; 5 μL of stable cell line suspension was injected into each group with a total of 1 × 10^6^ cells. The injection needle was held in position for 10 min and then slowly removed. Finally, the scalp was sutured.

### Hematoxylin–eosin (HE) staining

Brain slices were removed from the refrigerator, rewarmed at room temperature, and then washed three times with 1 × PBS for 5 min. The sections were stained with hematoxylin staining solution and observed repeatedly under a microscope for 7–8 min during the staining process. The brain slices were then removed and rinsed with running water for 20 min. The sections were then differentiated with a differentiation solution for 30 s and rinsed with running water for 15 min. Then, the sections were stained with eosin staining solution for 2–3 min and observed repeatedly under a microscope during the staining process. The brain sections were removed and rinsed with running water for 5 min. Then, the sections were successively dehydrated and cleared in 95% ethanol (I), 95% ethanol (II), 100% ethanol (I), 100% ethanol (II), xylene (I), and xylene (II) for 1 min each and then sealed with neutral gum.

### TUNEL assay and imaging

The animals were anesthetized and perfused with 0.1 M phosphate buffered saline and 4% paraformaldehyde. The fixed tumor tissues were extracted, embedded in paraffin, and cut into 5 μm sections. The sections were subjected to IHC or TUNEL staining, counterstained with 4′,6-diamidino-2-phenylindole (DAPI), and observed under a fluorescence microscope (Nikon, Kyoto, Japan).

### In vivo tumorigenesis in mice

Mice were randomized into the two groups. The animals were removed from the cervical vertebra and sacrificed after two and four weeks after implantation of cells, and tumor tissues were harvested, photographed, and weighed. Later, some tumors were placed in liquid nitrogen quick-freezing and stored in a refrigerator at − 80 °C for future use. All animal experiment procedures had been approved by the Medical Ethics Committee of Sichuan Provincial People’s Hospital (Approval Number: 2022-154).

### Immunofluorescence microscopy

Tissue immunofluorescence staining was performed using a series of 10-µm-thick sections (Leica Instruments). Cells were cultured in a complete medium containing 10% FBS and plated on slides coated with poly-D-lysine (PDL; Sigma-Aldrich, 5 ×). After 5 days, cells were fixed with 4% paraformaldehyde for 20 min at 4 °C followed by the permeabilization of cells for 10 min with 0.1% (vol/vol) Triton X-100. Cells were then blocked with 1% BSA solution in phosphate-buffered saline (PBS). Sections and cell climbing slices were incubated with the following primary antibodies: rat anti-Nestin (1;200, Abcam, MA, USA), rabbit anti-NeuN (1:200, Abcam, MA, USA), rabbit anti-GFAP (1:300, CST, MA, USA) or goat anti-Olig2 (1:200, Abcam, MA, USA) antibodies at 4 ℃ overnight. After washing with PBS, sections were then incubated with Alexa Fluor 555-conjugated chicken anti-goat IgG (1:1000; CST, MA, USA) antibody, Alexa Fluor 555-conjugated goat anti-rabbit IgG (1:1000; CST, MA, USA) antibody, and Alexa Fluor 488-conjugated goat anti-rat IgG (1:1000; CST, MA, USA). Finally, the coverslips were counterstained with 4′,6-diamidino-2-phenylindole (DAPI, Beyotime) and imaged with a confocal microscope (Zeiss LSM800, Germany).

### Western blotting

RIPA lysis buffer (Beyotime, Beijing, China) containing 1% protease and 1% phosphatase inhibitors was added to the lysed cells. Then, a BCA kit (Vazyme Biotech, Nanjing, China) was used to determine protein concentrations from each group. A one-quarter volume of 5 × SDS loading buffer was added to each sample which was then heated for 5 min at 100 °C to denature proteins. Next, protein samples (25 μg per sample) were separated by 10–15% sodium dodecyl sulfate-polyacrylamide gel electrophoresis. Once separated, proteins were transferred to a PVDF membrane, which was then blocked in 5% skimmed milk at room temperature (RT) for 1 h. Next, the PVDF membranes were incubated with primary antibodies at 4 °C overnight. The next morning, the PVDF membranes were washed three times in 1% TBST and incubated with relevant secondary antibodies for 1 h at RT. Finally, the membranes were washed in TBST and imaged with a Tanon-5200 chemiluminescent imaging system (Tianneng Technology Co., Ltd., Shanghai, China).

### Statistical analysis

Data were analyzed and significance was determined by GraphPad Prism version 6.02 software. Results are shown as means ± standard error (SE). Statistical significance was evaluated by a two-tailed, unpaired Student ‘s t-test or by one-way analysis of variance (ANOVA) followed by the Bonferroni test for multiple comparisons; p values < 0.05 were considered significant.

## Results

### NSCs expressed high levels of linc-NSC when compared with ESCs

**Fig. 1:**
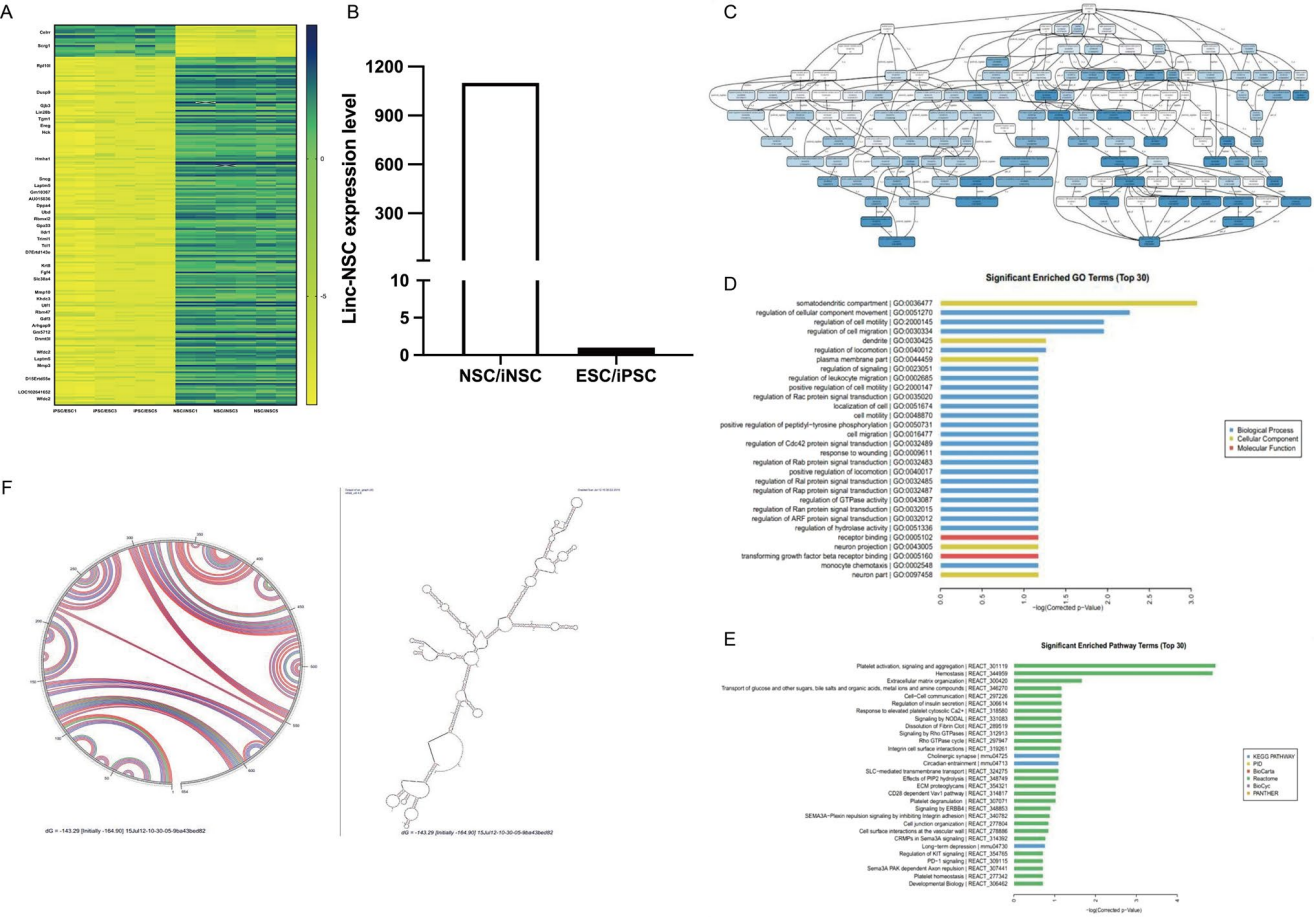
Prediction of candidate genes in NSC/ESC. **A** Heatmap map of predicted new lncRNAs in NSC/iNSC and ESC/iPSC. **B** Linc-NSC differential expression. **C** and **D** GO analysis (E) KEGG analysis. **E** The structures shown were predicted by Mfold

The global gene expression profiles of ESCs and NSCs were determined by Agilent mRNA expression arrays (n = 3/group). The Agilent mRNA expression array was performed by the CapitalBio Company (Beijing, China). After screening, two datasets (ESC/iPSC and NSC/iNSC) were used to evaluate the expression levels of linc-NSC (Fig. 1A). According to our analysis, linc-NSC was expressed at levels that were more than 1000-fold higher in NSC/iNSC when compared with ESC/iPSC (Fig. 1B). Next, we investigated the correlation between linc-NSC and mRNA expression levels in ESCs and NSCs. Analysis revealed a significant negative correlation between linc-NSC and 594 mRNA transcripts (r < −0.99, p < 0.05), while 371 genes exhibited a significant positive correlation with linc-NSC (r > 0.99, p < 0.05). Furthermore, two pluripotency-related genes, Oct4 (r = −0.9926) and Nanog (r = −0.9924), exhibited significant negative correlation with linc-NSC (p < 0.05). In addition, several genes, including MAP2 (r = 0.9953), DENR (r = 0.9901), GABAB_2_ (r = 0.9925), and S100B (r = 0.9966), which are known to be involved in neurogenesis, exhibited a statistically significant positive correlation with linc-NSC (p < 0.05; Fig. 1D). The gene expression profile data of stem cells was processed using Kobas (KEGG ontology-based annotation system) software. This facilitated the analysis GO function and signal pathways of linc-NSC, as well as the construction of a co-expression network for linc-NSC and differential mRNA. Notably, the significantly enriched GO functions included synaptic septum (GO: 0036477), the regulation of cell movement (GO: 0051270), and receptor binding (GO: 005102). The pathways that exhibited significant enrichment were platelet activation, signal transduction and aggregation (react: 301,119), extracellular matrix formation (react: 300,420), and cholinergic synapse (mmu04725) (Fig. 1E–F). We predicted the secondary structure of linc-NSC using a variety of methods (sequence alignment, lattice diagram, free energy, covariance model, and base pair maximization) (Fig. 1G) and detected a variety of stem rings or hairpin rings in the secondary structure of linc-NSC, which may be related to the regulation of gene transcription and binding to target molecules.

Thus, GO and KEGG analysis suggested that the linc-NSC may be negatively related to stem cell pluripotency and positively related to neurogenesis (Fig. 1C–E). The secondary structure of linc-NSC was predicted by Mfold software (Fig. 1F). We found a variety of stem rings or hairpin rings in the secondary structure of linc-NSC, which may be related to the regulation of gene transcription and binding to target molecules.

### Isolation and identification of NSCs and modified NSCs

**Fig. 2:**
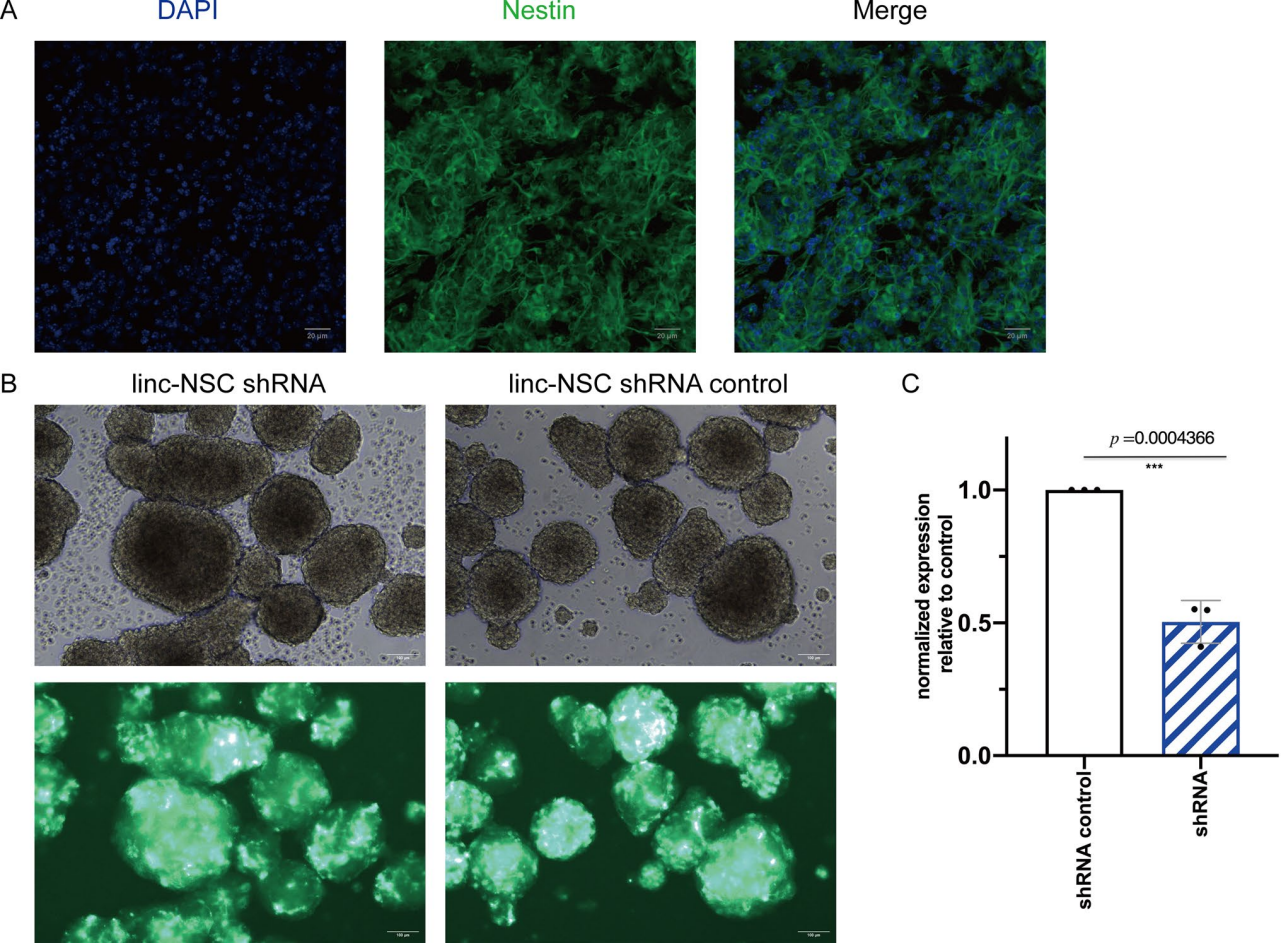
Identification of the NSCs and modified NSCs. **A** Using Nestin (green) as a marker we identified NSC in 2D adherent culture. **B** After 72 h, fluorescence microscopy was used to detect transfection efficiency, and the transfection efficiency was > 60%. The infected cells were then selected in medium with 1ug/ml of puromycin for 3 days. Scale bar = 100 µm **C** Linc-NSC expression was measured by qPCR to assess the knockdown efficiency of linc-NSC (n = 3, one-way ANOVA with post hoc test, ***P < 0.001)

NSCs were extracted from mouse fetuses on days 13–15, and the expression of Nestin was identified by immunocytochemistry in the isolated cells (Fig. 2A). The experimental cells were then segregated into two groups: a negative control sample group (shRNA control group) and a sample group infected with lentiviral interference vector (shRNA group). Gradually increasing concentrations of puromycin (0, 1, 2, 4, 8, 10 µg/mL) were administered to each group. After six days, the groups were subjected to microscopic observation, and the minimum lethal concentration, which resulted in the death of all NSCs but allowed for survival after transfection, was identified as the optimal screening concentration. Consequently, a dosage of 1 µg/mL was utilized to screen for lentivirus-infected NSCs (Fig. 2B). The knockdown of linc-NSC was confirmed by real-time qPCR, which demonstrated effective knockdown by approximately 50% (Fig. 2C, p < 0.001).

### The knockdown of linc-NSC promoted the proliferation and inhibited the differentiation and apoptosis of NSCs in vitro.

**Fig. 3:**
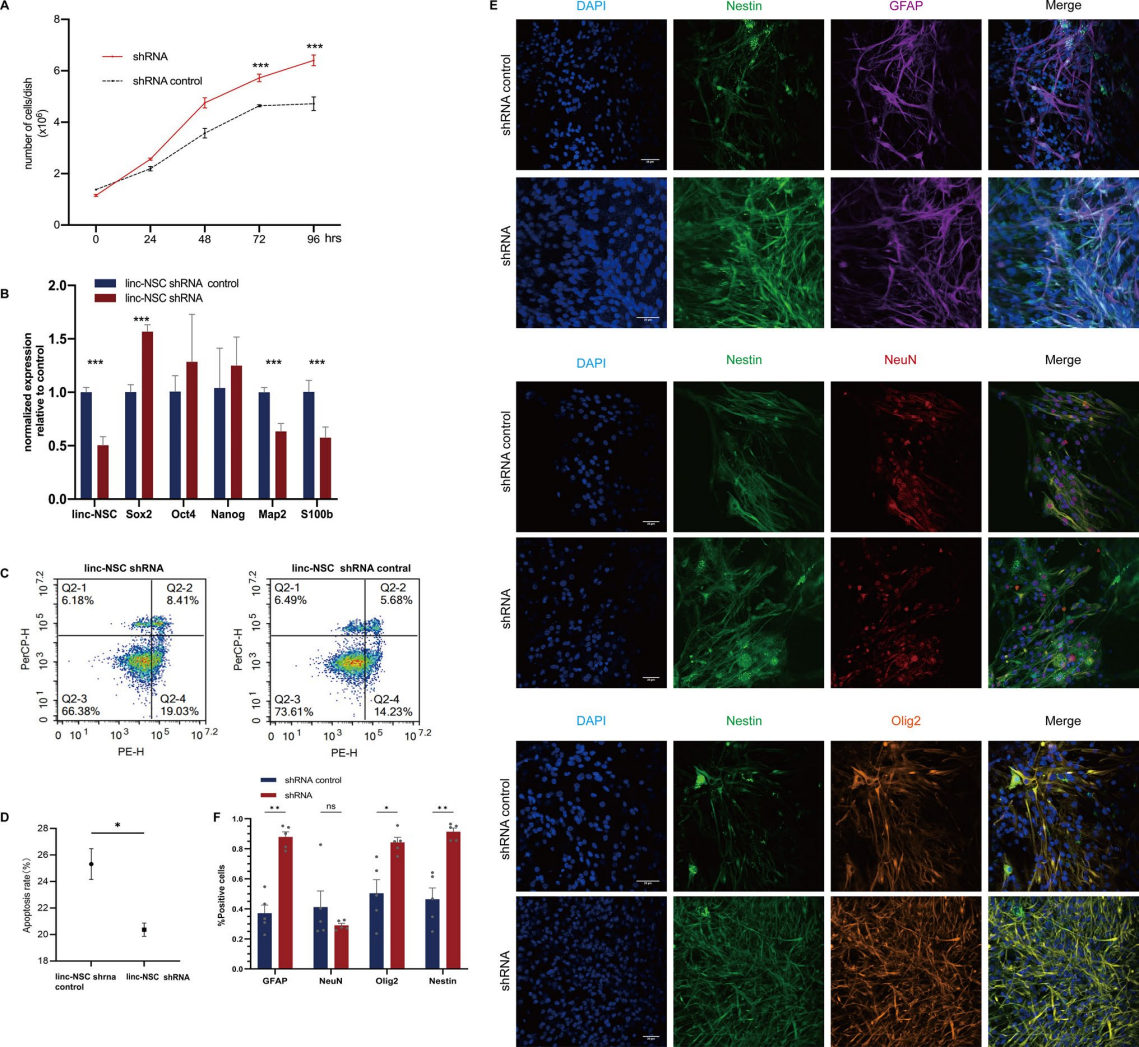
Linc-NSC knockdown promotes the proliferation and inhibits the differentiation and apoptosis of NSCs in vitro. **A** Cell doubling time studies in shRNA group and shRNA control group cells. The shRNA cells had a population doubling time of less 24 h and the shRNA control cells of about 72 h. **B** Stemness and neurogenic properties was evaluated by the expressions of three pluripotency genes (Sox2, Oct4, and Nanog) and two neurogenic related gene (Map2 and S100b) after knockdown linc-NSC. An asterisk indicates that the difference between controls and Sox2 is significant. **C** and **D** Flow cytometric analysis of single-cell suspension prepared from Accutase-digested sphere. The apoptosis rate was defined as the early apoptosis rate plus the late apoptosis rate. **E** and **F** Image of the neurosphere in adherent culture immunostained with Nestin and differentiation marker (GFAP, NeuN and Olig2). Green color, Nestin staining; purple, GFAP staining; red, NeuN staining; orange, Olig2 staining; blue, DAPI. Scale bar = 20 µm. (n = 5, Student’s t-test, *p < 0.05, **p < 0.01, ***p < 0.001)

The objective of this study was to investigate the impact of linc-NSC on the proliferation, differentiation, and apoptosis of NSCs. Following the establishment of cell cultures, cells were subjected to either suspension (SCs) or adherent (differentiated cells) conditions to assess proliferation and differentiation, respectively. The proliferation capacity of cells was evaluated by means of a cell growth curve, which involved cell counting. Analysis indicated that the doubling time was reduced in transfected shRNA cells (approximately 48 h) when compared to the shRNA control group (approximately 72 h) (Fig. 3A). To investigate the impact of linc-NSC knockdown on NSC stemness, we evaluated stemness gene expression and neurogenic properties in vitro under specific differentiating conditions (Fig. 3B). No statistically significant differences were observed in terms of Oct4 and Nanog expression between the two conditions. These findings suggest that linc-NSC promotes NSC differentiation. Additionally, an apoptosis assay was performed using the Annexin V-PE detection kit. Our analysis revealed that the shRNA group exhibited a significantly lower rate of apoptosis (p < 0.05) when compared to the shRNA-control group, which may be attributed in part to the down-regulation of linc-NSC, as evidenced by the results presented in Fig. 3C and D. Differentiation cultures of both shRNA and shRNA-control NSCs were conducted using 10% fetal bovine serum, and the cells demonstrated the ability to undergo tri-lineage differentiation (GFAP + , NeuN + , and Olig2 +) in vitro. However, the differentiation potential of the shRNA group was comparatively less pronounced than that of the shRNA-control group, as indicated by the statistically significant difference observed in Fig. 3E and F (p values in the figure legend).

### The overexpression of linc-NSC inhibited the proliferation of ESCs in vitro but promoted apoptosis

**Fig. 4:**
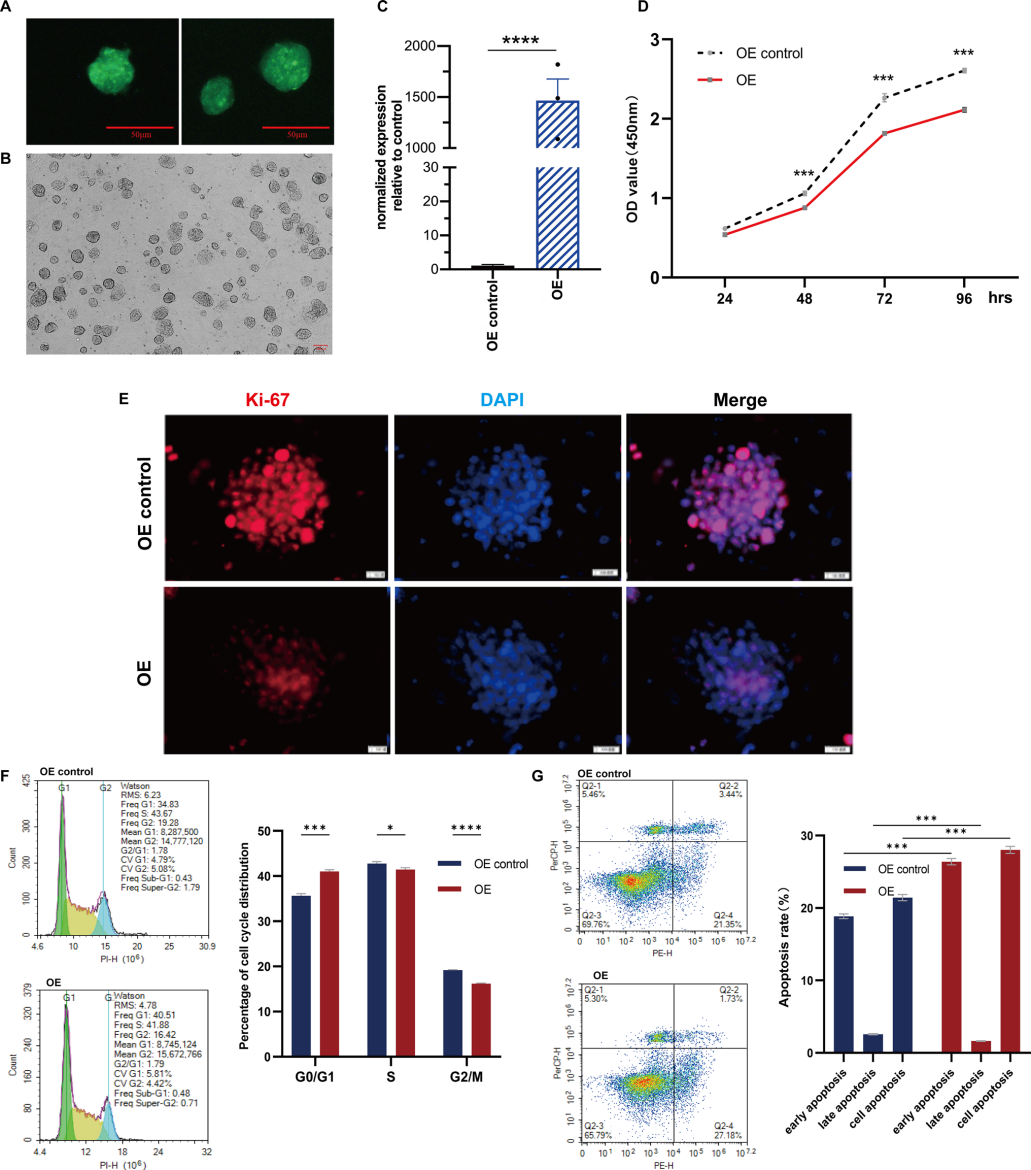
Linc-NSC inhibited the proliferation and promoted apoptosis of ESCs in vitro. **A** The normal embryonic stem cells (ESC) were subjected to stable transfection with green fluorescent protein (GFP). Scale bar = 50 µm. **B** The ESCs were sub-cultured on mouse embryonic fibroblasts (MEFs) as feeder cells in embryonic stem cell medium. Scale bar = 100 µm. **C** The transfection efficiency of linc-NSC was assessed by measuring Linc-NSC expression using quantitative polymerase chain reaction (qPCR). (n = 3 qPCR replicates). **D** Cell doubling time studies were conducted on the OE group and OE control group cells, with cellular viability analyzed using the CCK8 assay (n = 5 duplicates). The OE group cells exhibited a population doubling time of less than 40 h, while the OE control cells had a population doubling time of approximately 36 h. **E** Ki67 expression in the OE group and OE control group was evaluated through immunofluorescence staining. Scale bar = 100 µm. **F** The cell cycle was analyzed through flow cytometry, with statistical results presented. (n = 3, Student’s t-test, *p < 0.05, ***p < 0.001, ****p < 0.0001). **G** Apoptosis detection was conducted through flow cytometry, with statistical results reported (n = 3, Student’s t-test, ***p < 0.001)

We harvested cells from the overexpression control group (OE control) and overexpression (OE) linc-NSC group, and analyzed three samples from each group. The percent of GFP fluorescence expression exceeded 80% in both groups (Fig. 4A). Following transfection, the mESCs retained their typical morphology, characterized by island or nest growth, smooth and intact edges, strong refraction, and robust proliferation ability (Fig. 4B). The verification of gene expression was conducted by RT-qPCR, which yielded results indicating that the expression levels of linc-NSC in the OE group was over 1000-fold greater than that of the OE control group (Fig. 4C). This finding suggested that the construction of a stable gene overexpression cell line had been successful. Furthermore, CCK-8 assays demonstrated that the OE control group exhibited a significant increase in viability and cell proliferation rate (p < 0.01) (Fig. 4D). The immunofluorescence staining of Ki-67 were consistent with those of the CCK-8 assay (Fig. 4E). Detection of the cell cycle was accomplished by using propidium iodide (PI), a fluorescent dye that binds to double-stranded DNA, thus resulting in fluorescence. Subsequently, flow cytometry was employed to quantify the DNA content of cells. Cell cycle analysis was conducted based on the distribution of DNA content. Our findings indicated that the G1 phase was extended, the S phase remained unaltered, and the G2/M phase was reduced in the OE group in comparison to the OE control group (Fig. 4F). It is commonly observed that embryonic stem cells (ESCs) exhibit a short G1 phase and a high proportion of cells in S phase. The findings of the present study indicated that ESCs in the OE group exhibited an extended G1 phase and cell cycle arrest in the G1 phase, resulting in a negative impact on ESC proliferation. The application of annexin-V and PI staining allowed for the differentiation of early apoptotic (annexin-V + /PI −) and late apoptotic cells (annexin-V + /PI +). The OE group demonstrated a significantly higher overall rate of apoptosis in comparison to the OE control group, with a greater proportion of ESCs undergoing early apoptosis and a lower proportion undergoing late apoptosis in the OE group (Fig. 4G). In summary, the ESCs within the OE group exhibited a lengthened G1 phase and a shortened G2/M phase, in addition to an increased rate of apoptosis when compared to the OE control group.

### linc-NSC inhibited stemness maintenance and promoted the neurogenic differentiation capabilities of ESCs in vitro

**Fig. 5:**
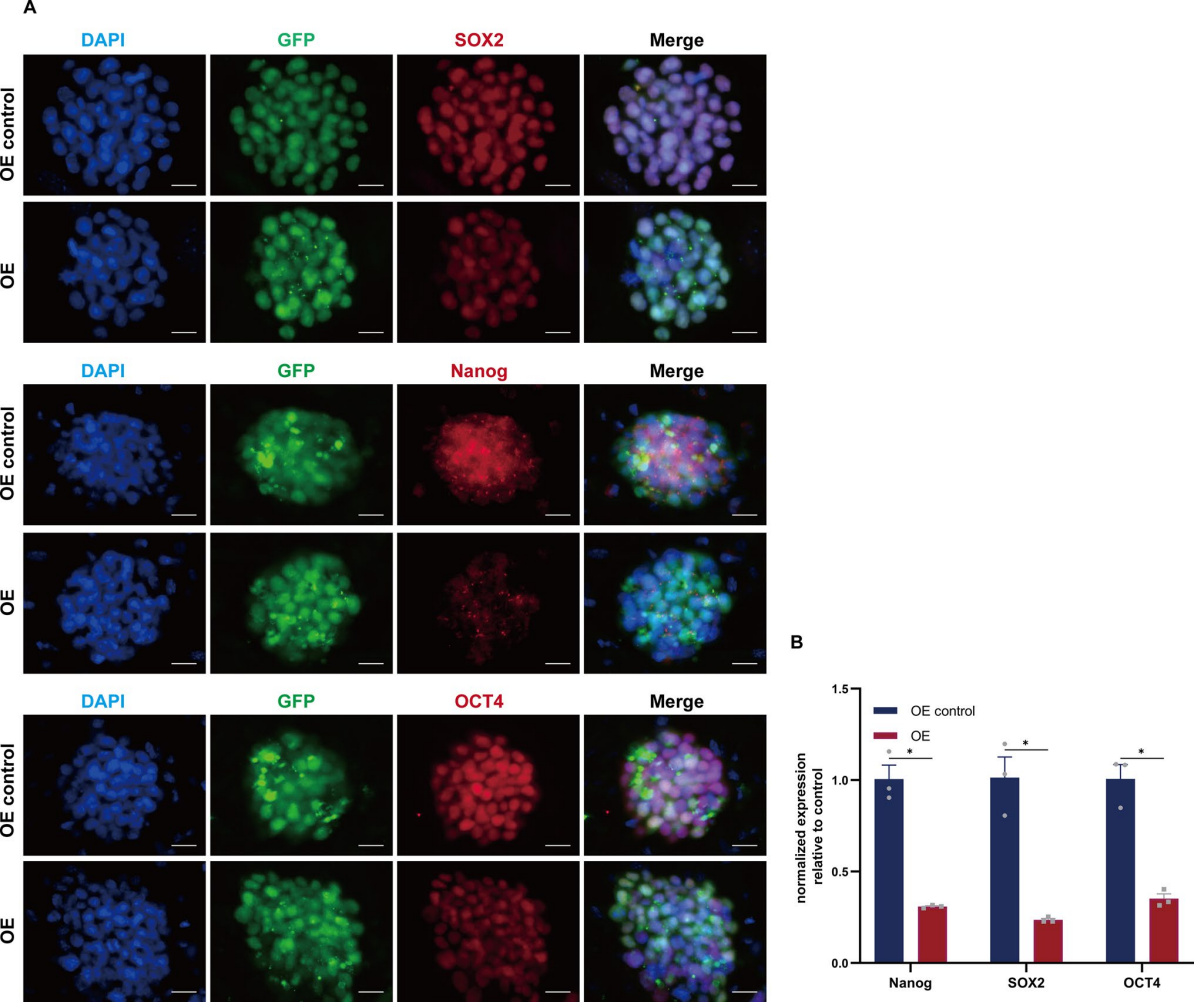
Linc-NSC inhibited the stemness maintenance of ESCs in vitro. **A** Immunofluorescence staining for the stem cell pluripotency markers Sox2 (red), Nanog (red) and Oct4 (red). Scale bar = 100 µm. **B** The statistical analysis of immunofluorescence (n = 3, Student’s t-test, *p < 0.05)

Both ESCs and iPSCs possess the capacity to differentiate into all cell types of the three germ layers, a unique attribute stemming from their pluripotency. Pluripotency is a fundamental characteristic of ESCs, with Nanog, Oct4, Sox2, Klf4, and other pluripotency-related genes playing a crucial role in this process. Induced pluripotent stem cells (iPSCs) can be successfully generated by the over-expression of transcription factors, including Oct4, Sox2, Klf4, and c-Myc, in mouse fibroblasts. The confocal microscopic images presented in Fig. 5A demonstrate that the overexpression of linc-NSC significantly reduced the fluorescent intensity of pluripotency genes (Nanog, Oct4, and Sox2) in most of the ESCs, while a weaker signal was observed in the control group. The results of RT-qPCR analysis, as depicted in Fig. 5B, were consistent with the immunofluorescence staining, indicating that the levels of Nanog, Oct4, Sox2, and Klf4 in the OE group were significantly lower than those in the OE control group.

**Fig. 6:**
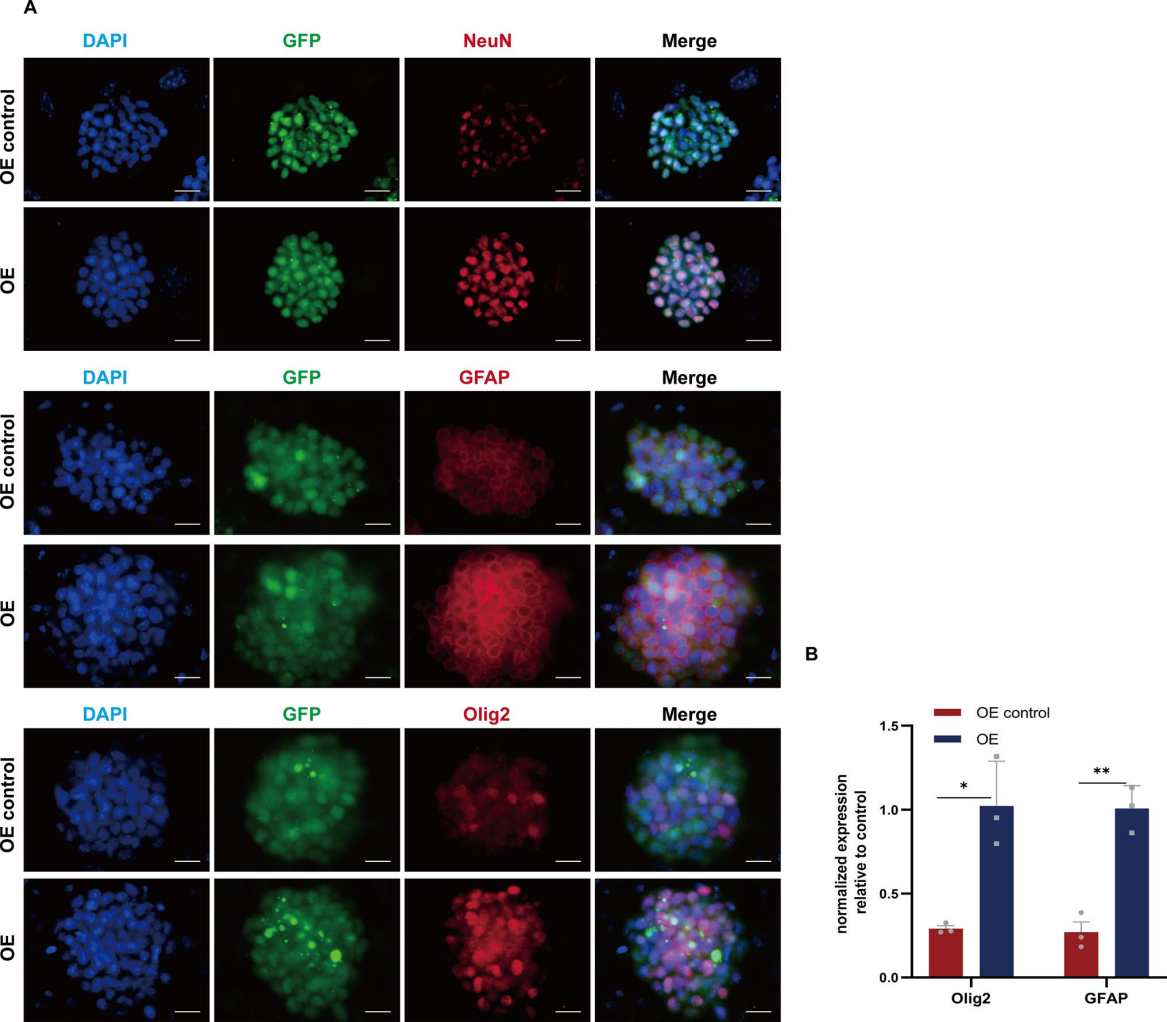
Linc-NSC promoted the neurogenic differetiation capabilities of ESCs in vitro. **A** Immunofluorescence staining for the stem cell differentiation markers NeuN (red), GFAP (red), and Olig2 (red). Scale bar = 100 µm. **B** The statistical analysis of immunofluorescence (n = 3, Student’s t-test,*p < 0.05, **p < 0.01)

The CNS is composed of two distinct cell types, namely neurons and glial cells, with the latter primarily consisting of astrocytes, oligodendrocytes, and microglia. In order to investigate the impact of linc-NSC overexpression on neural markers and RNA expression, immunofluorescence staining was conducted on several neural markers, including NeuN, Olig2, and GFAP. Our objective was to elucidate the potential effects of linc-NSC on neurogenesis. Both the OE control and OE groups were seeded in 24-well plates in equal numbers. Following a 2-day culture period (DMEM/F12, 50 ng/ml NGF,2uM Retinoic Acid, 2% B27,1% penicillin–streptomycin), immunofluorescence staining was conducted on ESCs using antibodies targeting NeuN, Olig2, and GFAP; the resulting staining patterns were visualized using a confocal microscope (Fig. 6A). Immunofluorescence staining revealed that the fluorescence intensity of NeuN, Olig2, and GFAP was notably stronger in the OE group when compared to the OE control group. Subsequently, RT-qPCR was performed to assess RNA expression levels, which demonstrated that the expression levels of NeuN, Olig2, and GFAP were also significantly higher in the OE group (Fig. 6B). In summary, these findings suggest that linc-NSC has the potential to enhance neurogenesis.

### linc-NSC enhanced the survival effect of NSC transplantation under the premise of non‐tumorigenicity and suppressed the stemness properties of NSCs

**Fig. 7:**
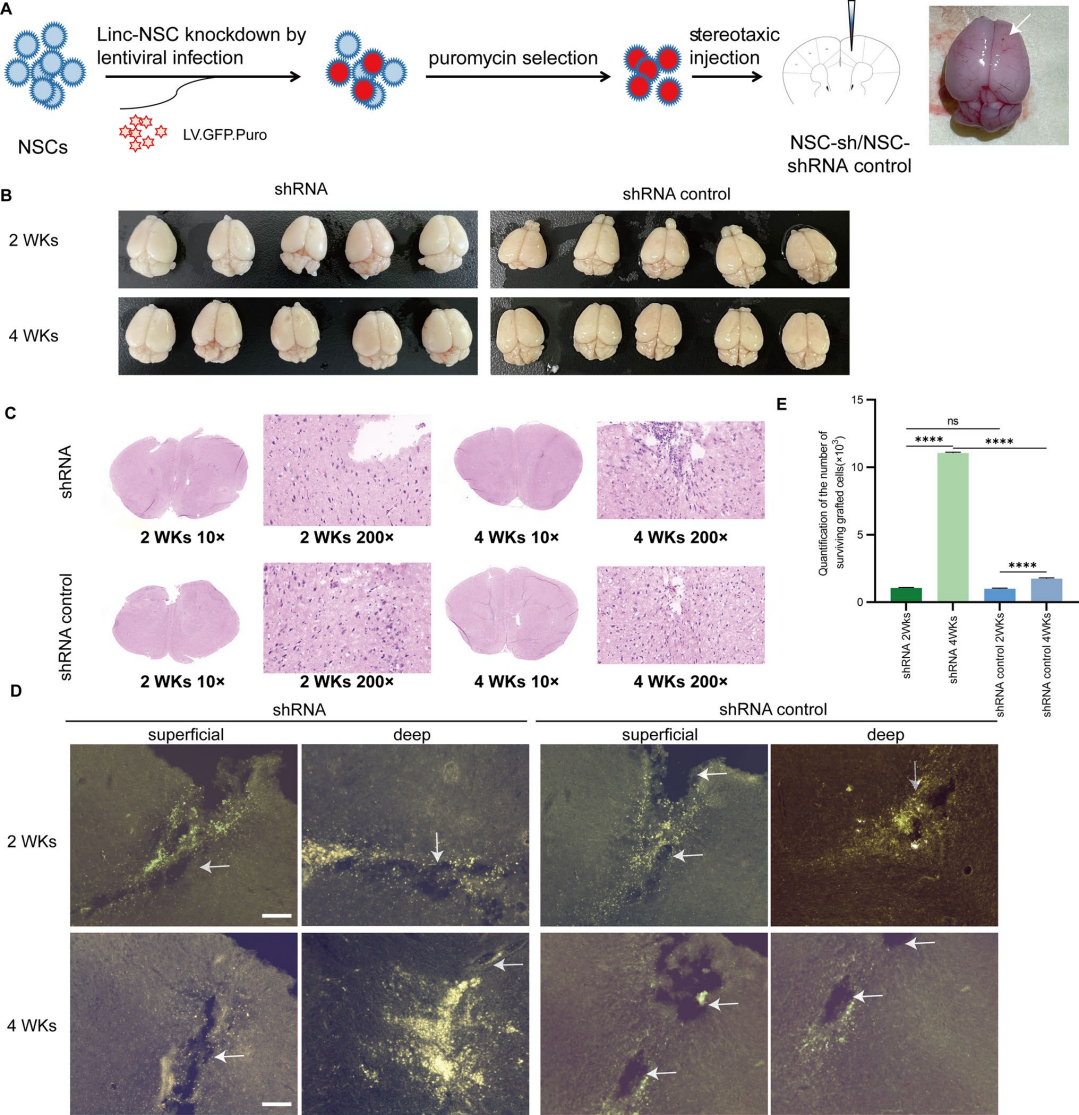
Linc-NSC enhanced the survival effect of NSC transplantation under the premise of non‐tumorigenicity. **A** A schematic depiction of the experiment conducted is presented. **B** The transplanted mouse brain's gross appearance is illustrated, and no macroscopic gross tumor formation was observed across all groups. **C** The explanted grafts were subjected to HE staining at two weeks (× 10, × 200) and four weeks (× 10, × 200) post-implantation. **D** The presence of GFP-labeled cells was detected through green fluorescent imaging of consecutive sections, with the injection needle tract indicated by an arrow. Scale bar = 100 µm. **E** The number of surviving grafted cells was quantified (n = 5, Student’s t-test, ****p < 0.0001, no statistically significant difference denoted by "ns")

**Fig. 8:**
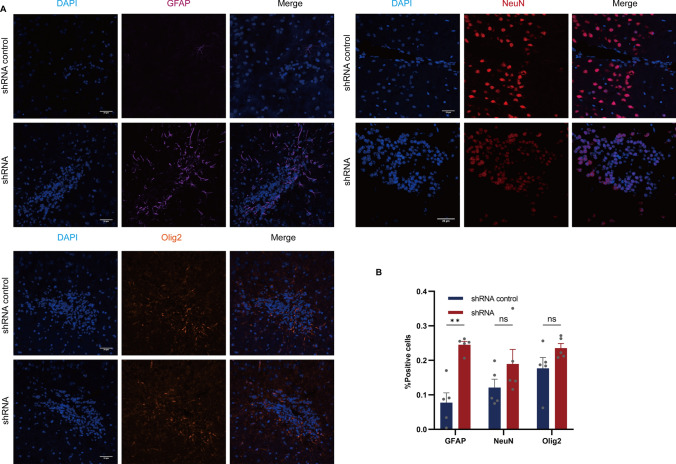
Linc-NSC suppressed the stemness properties of NSCs. **A** High power magnification of grafts two weeks and four weeks post-transplantation revealed immunofluorescence staining of GFAP (purple), NeuN (red), and Olig2 (orange). Scale bar = 20 µm. **B** The rates of GFAP + , NeuN + , and Olig2 + cells were subjected to statistical analysis between the shRNA and shRNA control groups (n = 5, Student’s t-test, **p < 0.01, no statistically significant difference denoted by "ns")

Fifteen mice per group were sacrificed at 2 weeks and 4 weeks post-grafting to evaluate the survival and condition of the transplanted cells (Fig. 7A). All animals survived the study period up to 4 weeks post-transplantation. The survival of grafted NSCs was assessed by detecting GFP-positive under a 40 × magnification objective 4 weeks after transplantation. No tumor formation was observed 2 weeks after cell transplantation, and transplanted cells survived in each group (Fig. 7B and C). After a duration of 4 weeks, the quantity of GFP + NSCs in the shRNA group exhibited a statistically significant increase in comparison to the NSCs in the shRNA control group, while maintaining non-tumorigenicity (Fig. 7B and C, p < 0.001). However, the number of viable cells declined with an increase in the duration of transplantation in the shRNA-control group, and the presence of transplanted cells was not observed in every animal. In contrast to the grafts surrounding the transplanted tissue in the shRNA control group, the overall density of GFP-positive cells within the grafted tissue was significantly different (Fig. 7D and E, p = 0.180 and p = 0.018, respectively) when compared to the transplant in shRNA hosts. The NSCs were cultured as spheres in vitro, dissociated onto laminin-coated coverslips, and were induced to differentiate. These findings indicate that the knockdown of linc-NSC has the potential to prevent apoptosis and promote cell survival. Additionally, we evaluated the differentiation capacity of shRNA control cells following animal transplantation. The in vivo differentiation analysis revealed that these cells were more proficient in generating GFAP + cells when compared to shRNA cells. However, there was no significant difference observed in the generation of NeuN + and Olig2 + cells (Fig. 8A and B).

### linc-NSC mitigated the tumorigenicity of ESCs transplantation by promoting differentiation

**Fig. 9:**
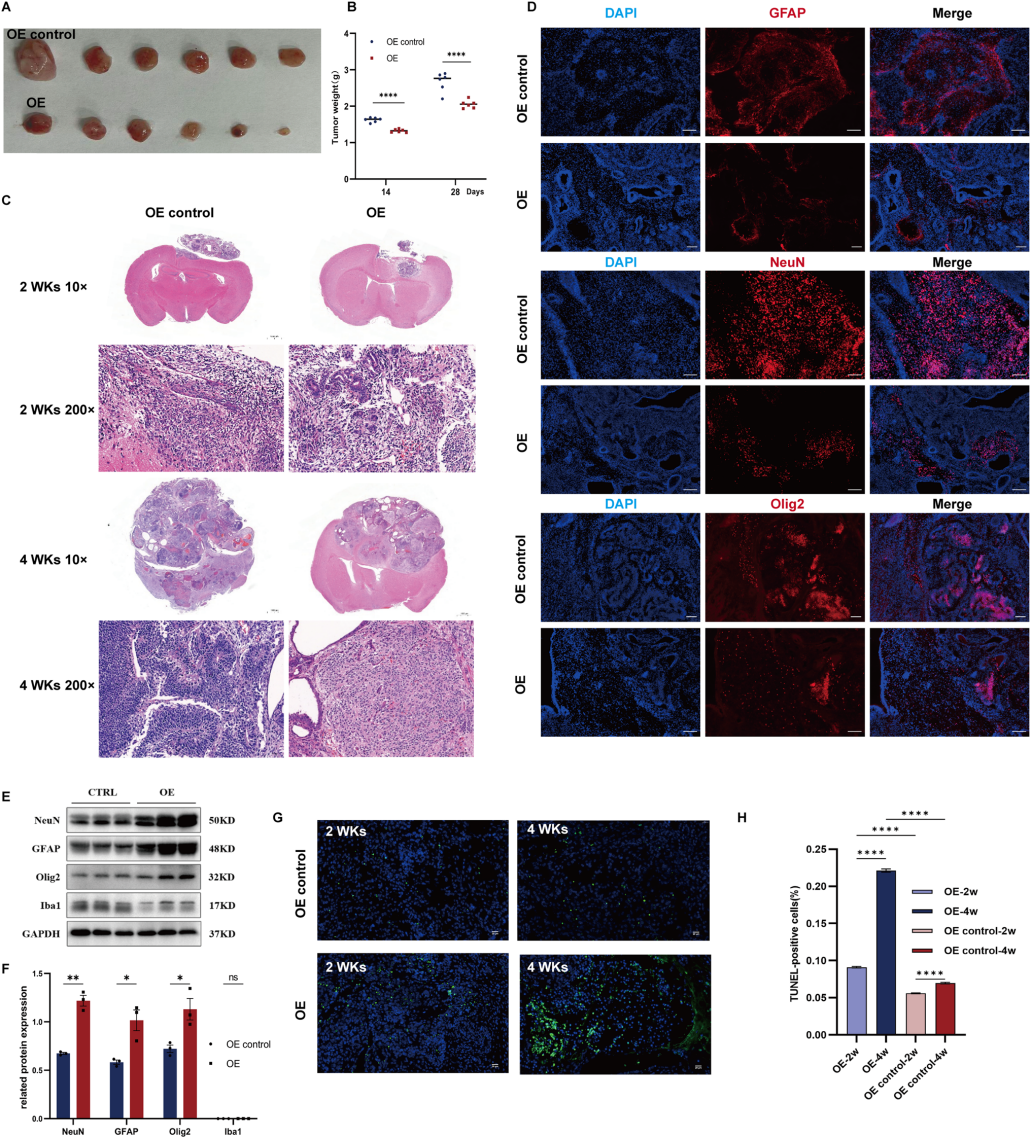
Linc-NSC mitigated the tumorigenicity of ESCs transplantation by promoting differentiation. **A** and **B** Tumors were collected from the sacrificed mice, and the size of tumors was compared. **C** HE staining for the tumor formed 2 and 4 weeks (× 10, × 200) after cell transplantation. Scale bars = 1000 μm (left) and 50 μm (right). **D** Effect of linc-NSC on neurogenesis (GFAP + , NeuN + , and Olig2) of tumor tissue 28 days after cell transplantation. Scale bars = 100 μm. **E** Western blot assay of GFAP and NeuN in brain tumor tissue formed 28 days after cell transplantation. **F** Statistical graph of relative protein expression. (n = 3, Student’s t-test, *p < 0.05, **p < 0.01, no statistically significant difference denoted by "ns"). **G** TUNEL staining. Scale bar = 20 μm; DAPI: blue, TUNEL: green. **H** Quantitative analysis of TUNEL-positive cells (n = 5, one-way ANOVA, ****p < 0.0001)

Following cell transplantation, 12 mice from both the OE control group and the OE group were weighed and assessed for brain tumor size after 2 weeks. At 14 days and 28 days post-transplantation, three mice from each group were perfused and fixed, and the entire brain tissue was subjected to HE staining to identify the tumor type. We conducted a comparative analysis of brain tumor size and weight between the two groups, 14 days after cell transplantation. Analyses indicated that both groups exhibited significant differences in tumor size and weight, with the OE group demonstrating larger and heavier tumors than the OE control group (Fig. 9A and B, p < 0.0001). Histological examination by HE staining revealed that tumor cells in both groups were poorly differentiated (Fig. 9C). Notably, there were no significant differences in tumor cell morphology between the two groups, with cells arranged in single or multiple layers, exhibiting varying nuclear sizes and heterogeneity (Fig. 9C).

On the 28th day following cell transplantation, the immunofluorescence results of GFAP + , NeuN + , and Olig2 + staining indicated that the OE group exhibited a greater fluorescence intensity than the OE control group (Fig. 9D). These findings were consistent with the results obtained from immunoblotting (Fig. 9E and F). TUNEL assay and DAPI staining revealed a co-localisation of staining in the nucleus, which indicated the occurrence of apoptosis. TUNEL staining showed a large increase in apoptotic positive cells in OE group in vitro (Fig. 9G and H, p < 0.0001, 2 week; p < 0.0001, 4 week).

## Discussion

One of the primary challenges associated with the transplantation of SCs pertains to the regulation of stem cell survival, proliferation, and differentiation post-transplantation. Despite the potential efficacy of this approach, the occurrence of significant grafted-cell death and limited tissue repair capacity may hinder its overall effectiveness. While recent efforts have focused on enhancing the survival rate of stem cells in vivo, further improvements are necessary to optimize their survival and repair capabilities [19]. The current investigation revealed that ESCs exhibit tumorigenicity while NSCs do not. Additionally, early sequencing analysis has identified a highly expressed, yet unknown, segment of long intergenic non-coding RNA (lincRNA) on chromosome 16 in NSCs without tumorigenicity. As a result, the primary objective of this study was to investigate the potential correlation between the elevated expression of lincRNA in NSCs and tumorigenicity. Surprisingly, our findings indicated that this particular lincRNA was closely associated with enhancing the survival, proliferation, and differentiation of transplanted NSCs, while simultaneously reducing apoptosis.

Long non-coding RNAs (lncRNAs) have driven research focused on their effects as oncogenes or tumor suppressors [20]. Stem cells and tumor cells share common mechanisms with regards to regulating proliferation, self-renewal and signal transduction [21]. However, the functions and mechanisms of most lncRNAs in the transplantation of NSCs/ESCs remain unclear.

The impediments to transplantation are attributed to the loss, death, or tumor formation of grafts. LincRNA, an RNA molecule transcribed by non-coding sequences situated between coding protein genes, was previously disregarded due to its lack of direct protein encoding capacity. However, recent research has revealed that these molecules can exert regulatory effects at the genetic, epigenetic, transcriptional, and translational levels. Moreover, lincRNA can interact with a broad spectrum of cellular molecules involved in diverse cellular activities. The genome contains a significantly higher proportion of long intergenic non-coding RNA (lincRNA) compared to messenger RNA (mRNA) and small molecule RNA, including microRNA (miRNA), small interfering RNA (siRNA), and Piwi-interacting RNA (piRNA). Due to the relatively conserved sequence of lincRNA, its structure and function are more intricate, making the investigation of its role and mechanism a prominent area of research [12]. Currently, the identified roles of lincRNA primarily encompass the regulation of gene expression through cis- or trans-regulatory mechanisms, the modulation of RNA splicing and degradation, the provision of a scaffold for nuclear materials, and functionality as a housekeeping gene or molecular "sponge" in the form of small molecule RNA [22]. These aforementioned mechanisms entail transcription occurring in the promoter region upstream of the gene that encodes the protein, the hindrance of neighboring gene expression, binding to the transcript of a protein-encoding gene, and the creation of endogenous siRNA through the activity of Dicer enzyme to govern gene expression. Additionally, microRNAs can induce gene silencing by attaching to mRNAs and competing with endogenous RNAs (ceRNAs) to regulate gene expression through competitive binding with microRNAs. Furthermore, ceRNAs have the ability to bind to microRNAs via response elements (MRes), thereby inducing gene silencing, a crucial RNA-RNA regulatory pathway with significant biological implications [23–26]. LncRNAs, which can span thousands of nucleotides, serve as ideal substrates for the adsorption and binding of miRNAs and compete for the occupation of a large number of miRNAs within the cell, acting as sponges to buffer their effects and impair their ability to interfere with the mRNA encoding protein of target genes. This regulatory mechanism of the lncRNA-miRNA mutual ceRNA relationship is essential for the early multilineage differentiation of NSCs, which determines the differentiation direction and proliferation ability of NSCs.

The transcription factor Sox2 is a critical component in the preservation of adult NSC stemness. Over the last two decades, SCs have been shown to be significantly involved in cancer and are instrumental in tumor formation and pathogenesis. The delicate interplay between NSC self-renewal and differentiation is essential for appropriate neural development. To mitigate the risk of teratoma formation, the transplantation of differentiated stem cells is preferable to undifferentiated stem cells. However, differentiated cells lack the ability to proliferate and repair. Thus far, the precise mechanisms responsible for the proliferation and differentiation of NSCs has yet to be elucidated. Neither growth factor stimulation nor transplantation can effectively regulate the proliferation and differentiation of NSCs and differentiate into the expected cells; the mechanisms controlling differentiation are regulated by multiple factors. NSCs undergo lateral differentiation and reverse differentiation phenomena. Clarke et al. transferred Lac-Z labeled mouse NSCs into the blastocysts of chickens and mice; these underwent reversible transdifferentiation into three germ layer tissues, including the brain, heart, liver, intestine, and other tissues [27].

In vivo, miRNA can affect the expression of related genes by specifically binding to RNA, thus participating in the regulation of various cellular functions. The genes that can specifically bind to miRNA by sequence are referred to as the target genes of the miRNA. Compared with the linc-NSC shRNA control NSCs, transfected shRNA NSCs exhibited an increased survival ability. Moreover, it can preserve the proliferation and differentiation of cells without causing a teratoma. On the other hand, by overexpressing the expression of linc-NSC in ESCs, we found that the volume and quality of the tumor were reduced, although tumor formation was not completely inhibited. We believe that the factors or signal pathways that regulate the tumorigenicity of stem cells are not singular, and that linc-NSC may only affect a certain signal pathway, thus altering tumorigenicity. Several possible mechanisms may be involved. Under physiological or pathological conditions, cell proliferation, apoptosis, differentiation, and regeneration are strictly regulated by specific signaling transduction pathways. Previous research showed that the signaling pathway involved in promoting the proliferation of transplanted NSCs is largely dependent on the physiological activation of the Wnt pathway [28, 29]. The Wnt signaling pathway is crucial for stem cell survival, proliferation, differentiation, and maintenance [30]. In a previous study, Hsu et al. used GSK-3 inhibition to activate the Wnt/β-catenin pathway, thus enhancing the proliferation and expansion of neural progenitors while suppressing neuronal differentiation [31]. It is well-known that the aberrant activation of the canonical Wnt signaling pathway (or Wnt/β-catenin pathway) promotes tumorigenesis by regulating cell survival, proliferation, and invasion in many cancers [32]. We identified lncRNA and miRNA gene sequences by querying a relevant database. The specific binding sequences were calculated using Miranda software. To predict the target genes of interaction, we screened for binding sites with a score > 150 and an energy < -20 (Supplementary File 3, Figure A–G). We predicted that linc-NSC might affect the ceRNA network of linc-NSC-mmu-mir-7239-3p-Apc2. However, dual-luciferase reporter assays showed that mmu-miR-7239-3p could not bind to the 3'-UTR of linc-NSC and Apc2 (Supplementary File 3, Figure H).

We performed an in-depth literature search and found that the Wnt signaling pathway regulates the Hippo signaling pathway, which in turn influences the undifferentiated stem cell subpopulation involved in tissue replenishment and repair [33, 34]. Complex molecular mechanisms balance the proliferation, death, and fate of SCs. In particular, the number and activity of SCs need to be strictly monitored during development and regeneration to produce organs with predetermined sizes [35]. Mutations in the components of this pathway result in organ overgrowth due to increased mitosis and reduced susceptibility to cell death, most intuitively manifested by the formation of teratomas in transplanted stem cells. Evidence suggests that the Hippo pathway can modulate its effect on tissue size by directly regulating the proliferation and maintenance of SCs. Therefore, linc-NSC may promote the proliferation of NSCs by activating the Hippo pathway but this requires future investigation.

In summary, we first reported that linc-NSC, a completely unknown lncRNA fragment, was highly expressed in NSCs, and that the knockdown of linc-NSC predicted a better performance of NSCs in vitro and transplantation in vivo. Furthermore, by overexpressing the expression of linc-NSC in ESCs, we found that the volume and quality of tumors were reduced, although tumor formation was not completely inhibited. Functionally, linc-NSC induces apoptosis and reduces the proliferation of SCs. Our research will help to refine studies relating to the safety and effectiveness of transplanted SCs. Our data provide a novel target for gene manipulation therapy for patients requiring transplantation for SCs therapy, which may significantly improve the survival rate of transplanted cells without tumorigenesis.

### Supplementary Information

Below is the link to the electronic supplementary material.

Supplementary file1 (DOCX 109 KB)

Supplementary file2 (XLSX 10 KB)

Supplementary file3 (DOCX 966 KB)

## Data Availability

The data that support the findings of this study are available from the corresponding author upon reasonable request.
