# Supplementary material for: Linc-NSC affects cell differentiation, apoptosis and proliferation in mouse neural stem cells and embryonic stem cells in vitro and in vivo
Source: Cell Mol Life Sci. 2024 Apr 14;81(1):182. doi: 10.1007/s00018-024-05224-0 (PMC11016521; doi:10.1007/s00018-024-05224-0)
Supplement: Supplementary file 1 — Supplementary file1 (DOCX 109 KB) [file 18_2024_5224_MOESM1_ESM.docx]

**Supplementary File 1**


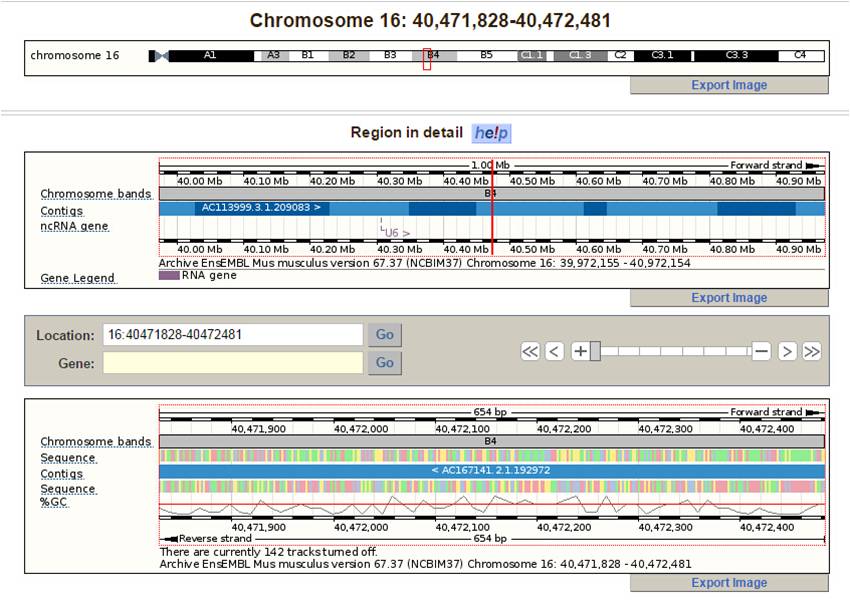


>16dna:chromosome chromosome:NCBIM37:16:40471828:40472481:1GGCTTTGAAAACTATAACGTCTTGATTATTAATGTACAAACAGACTACAAGATTTCTGAAATTATCAGTGATGGCTCCTAATTGTACTTCCATTTTACTTTCATGGGCCTTGTCCAAAGTAGGTACTAAAAGGCAAAGAAGAAAACCAGGAAGAAAGGACACAGTAGAGTTACTCAAAATGTCATACATATGTATGGGTCTTTCTTACTTCAAGTGAGAGCTACATAAATCCAAGGGCCTCTATCAAGCCCATAATCCTTGGTGACTACCTTTGATTGCGTGCTCATTCAAAACGCTGAGCACCCCATGGGAATGTGTTGAGAAAATTCCTAGCAGCCCCTGAGCAGAGCCATCAGAGCAAGAGAGCGAGAGAGCAAGAGCGCTCTTTTCTAGCAAGGGTGCTG**GGAAGTGAGGAGAGGGCTATT**TCTAGAGATCACATTGCAAGGGAGCTAATCAGACATTCCATGGGTAGTGACAACCAGCAATTTATTGTGATGCTTCTCAGCTCAAAGGAACAGCAGGGTGTGACAAGAACAGTTTGCTC**AGCTACACACAGACATCATG**GTTTACTCTTGGATTAAAAAAAAAAAATTAACCGTGCTTATTTTGGCAAAAAGAAAGAAGAAAAAAAGAAAAAAAAAAGAAACCTCTCAG

LOCUS X16 654 bp linear 10-JUL-2015DEFINITION dna:chromosome chromosome:NCBIM37:16:40471828:40472481:1.ACCESSION X16VERSION KEYWORDS .SOURCE Unknown. ORGANISM Unknown. Unclassified.FEATURES Location/Qualifiers source 1..654BASE COUNT 228 a 124 c 142 g 160 t
