# Supplementary material for: Linc-NSC affects cell differentiation, apoptosis and proliferation in mouse neural stem cells and embryonic stem cells in vitro and in vivo
Source: Cell Mol Life Sci. 2024 Apr 14;81(1):182. doi: 10.1007/s00018-024-05224-0 (PMC11016521; doi:10.1007/s00018-024-05224-0)
Supplement: Supplementary file 3 — Supplementary file3 (DOCX 966 KB) [file 18_2024_5224_MOESM3_ESM.docx]

First, we analyzed the potential miRNAs (using Miranda software to calculate the sequences that can specifically bind to each other, predict the target genes for interaction, and ultimately screen for binding sites with Score>150 and Energy<-20), and found the target mRNAs for potential miRNAs (intersecting with linc-NSC miRNAs to obtain the binding relationship between linc-NSC-miRNA-mRNA), Analyze the target mRNAs with significantly increased NSC expression compared to ESC (elevated in the NSC, as this is in line with the ceRNA regulatory mechanism), then intersect the target mRNAs and perform Go functional annotation (top 30 enriched GO terms) and KEGG analysis (top 30 enriched Path terms) to select mRNAs related to stem cell differentiation, neural cell differentiation, proliferation, apoptosis, etc. By listing the final target mRNAs and corresponding miRNAs (with conditions where miRNA binds to Linc-NSC and target mRNA, and both target mRNA and Linc-NSC are expressed significantly in NSC, only Linc-NSC-mmu miR-7239-3p-Apc2 may fulfill all of these criteria).

The pathway enrichment results showed that ceRNA of linc-NSC is enriched in the Wnt Signaling Path. The Wnt signaling pathway is a complex network of protein interactions, most commonly found in embryonic development and cancer, involved in regulating functions such as stem cell differentiation, neural cell differentiation, proliferation, and apoptosis. So the interaction between Linc-NSC, Apc2, and mmu miR-7239-3p was detected through dual luciferase assay. The first pair of 293T cells were co transfected with Linc-NSC, Apc2, and mmu miR-7239-3p: 1) Linc-NSC-WT+miR-NC; 2) Linc-NSC-WT+mmu miR-7239-3p;3) Linc-NSC-MUT+miR-NC; 4) Linc-NSC-MUT+mmu miR-7239-3p. The second pair: 1) Apc2-WT+miR-NC; 2) Apc2-WT+mmu miR-7239-3p; 3) Apc2 MUT+miR-NC; 4) Apc2 MUT+mmu miR-7239-3p cells Data was obtained by employing a Dual-luciferase reporter assay gene detection kit to facilitate the detection of dual luciferase. As shown in the Figure 1, the results of this dual luciferase detection can be determined as: mmu miR-7239-3p does not bind to the 3 'UTR of Linc-NSC and Apc2.


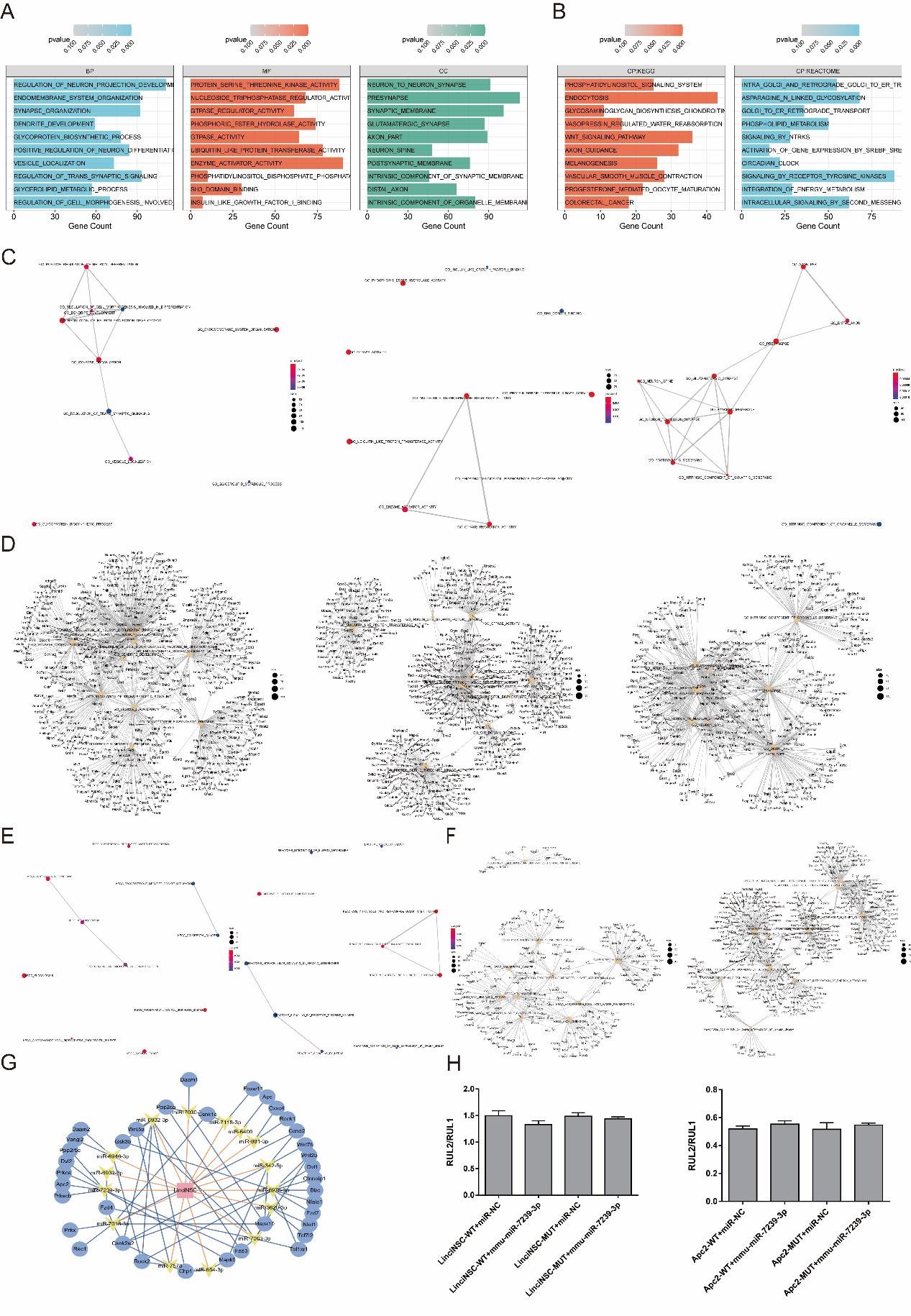


Fig1. (A)The GO database is utilized to annotate Gene Ontology (GO) terms from Biological Process (BP), Molecular Function (MF), and Cellular Component (CC) categories, thereby capturing all GO terms associated with genes. The Fisher test is employed to determine the statistical significance (P-Value) of each GO term, enabling the identification of GO terms with a high degree of gene significance. (B) The Fisher test was employed to compute the significance level (P-Value) of the Pathway in order to identify the Pathway Term with a high degree of gene significance. (C) The top 10 items in go term: GO term of BP, MF and CC in turn. (D) Network diagram of the relationship between GO term and differential genes Gene-Concept Network. (E) Pathway terms relational network diagram Enrichment Map. Top 10 entries in Pathway term: KEGG and REACTOME in turn. (F) Network diagram of Pathway term and differential genes Gene-Concept Network. Top 10 entries in Pathway term: KEGG and REACTOME in turn. (G) The results of pathway enrichment showed that ceRNA of lincNSC was enriched in Wnt Signaling Pathway. (H)Dual luciferase reporter assays.
